# VaxLab: integrated platform for rapid multistrategy mRNA vaccine design

**DOI:** 10.1038/s12276-026-01637-y

**Published:** 2026-04-08

**Authors:** Junsoo Kim, Yoojung Han, Chae Young Kwon, Hyeshik Chang

**Affiliations:** 1 Center for RNA Research, Institute for Basic Science, Seoul National University, Seoul, Republic of Korea; 2 Interdisciplinary Program in Bioinformatics, Seoul National University, Seoul, Republic of Korea; 3 School of Biological Sciences, Seoul National University, Seoul, Republic of Korea

**Keywords:** Drug development, Computational platforms and environments, RNA vaccines

## Abstract

mRNA vaccine efficacy depends on sequence optimization, but designing optimal sequences is challenging due to complex cellular RNA regulatory mechanisms. Here we present VaxLab, an open-source web platform that provides the complete design-to-synthesis workflow for mRNA vaccines in a unified interface. VaxLab incorporates four codon optimization algorithms based on distinct approaches: codon usage matching, secondary structure design and deep generative models. 5′ and 3′ untranslated regions can be designed using provided generative models or selected from a predefined sequence library. The designed sequences are then evaluated and reported for ten predictive metrics for RNA stability, protein expression and manufacturing risks. An integrated sequence editor with annotation tools enables further adjustments, and final sequences are exported in formats compatible with commercial DNA synthesis services. We validated VaxLab using influenza hemagglutinin sequences, completing the full design workflow in under 5 min per variant. In HCT116 cells, optimized variants showed up to 9.5-fold differences in protein expression, with most achieving at least 2.9-fold higher expression than the wild-type sequence. VaxLab provides an integrated optimization workbench that accelerates mRNA vaccine design by enabling principled selection among multiple optimization techniques informed by current understanding of RNA regulation.

## VaxLab streamlines mRNA vaccine design with advanced algorithms

mRNA vaccines have shown great promise, especially during the COVID-19 pandemic, due to their rapid development timelines and effectiveness. However, early versions needed high doses to be effective. This study addresses how to improve mRNA vaccine design to boost protein production and stability. Researchers developed VaxLab, a tool that optimizes mRNA sequences by combining multiple design algorithms. Using VaxLab, the team designed mRNA for influenza hemagglutinin, a key viral surface protein, and tested various optimization approaches. Some strategies significantly increased protein production while remaining practical to manufacture. Overall, the results demonstrate that VaxLab can streamline mRNA design process, offering a promising path toward faster, more effective mRNA vaccine development.

This summary was initially drafted using artificial intelligence, then revised and fact-checked by the author.

## Introduction

mRNA vaccines achieved strong clinical efficacy and rapid development timelines during the COVID-19 pandemic^1,2^. This success expanded both preventive and therapeutic opportunities in infectious diseases, oncology and rare genetic disorders^3–6^. Unlike attenuated and inactivated vaccines requiring viral culture, mRNA vaccines use in vitro transcription for rapid and scalable production^7^. However, early formulations required high doses to elicit adequate immunity due to insufficient optimization of formulation, delivery and protein/RNA sequence design^3^.

Codon selection optimization is essential for maximizing protein production from mRNA^8–10^. Traditional methods enhance translation by selecting synonymous codons that match usage patterns in highly expressed endogenous mRNAs, which have co-evolved with cellular tRNA abundance, while suppressing codons that translate inefficiently due to weak codon:anticodon pairing and other factors^11^. Recent discoveries show that highly structured mRNA exhibits superior stability in storage solutions and within cells, while also reducing innate immune responses^12–14^. These findings led to the development of two optimization tools: LinearDesign^15^ and RiboTree^14^. Both programs optimize coding sequences (CDSs) to minimize RNA folding free energy while maintaining codon preferences. LinearDesign uses dynamic programming to achieve deterministic optimization rapidly, whereas RiboTree employs Monte-Carlo tree search, which provides greater flexibility in objective function design.

Complementing these biophysical and structural prediction methods, machine learning approaches now leverage deep neural networks trained on codon usage patterns from highly expressed genes and large-scale experimental datasets^16–20^. CodonBERT by Ren et al.^20^ exemplifies this strategy through a BERT-based transformer model trained on human CDSs paired with ribosome profiling and mRNA half-life measurements. Such models efficiently search for optimized CDSs that maximize both RNA stability and protein production for a given protein.

Optimization of untranslated regions (UTRs) has advanced alongside CDS optimization to enhance ribosome recruitment, assembly and translation initiation^21–23^. Many mRNA drugs use UTRs from highly expressed genes, particularly human β-globin (*HBB*)^24^, which has evolved for constitutive, high-level protein expression with high protein output per mRNA molecule. Recently, synthetic UTR design strategies based on machine learning have emerged as alternatives to naturally derived sequences^23,25–29^. Predictive and generative models, trained on ribosome occupancy profiles of endogenous mRNAs or artificial reporters containing degenerative sequence patches, can generate optimized UTR sequences. For instance, GEMORNA-UTR uses a decoder-only transformer architecture to design both 5′ and 3′ UTRs, achieving up to 3-fold increase in protein expression compared with the UTR combination used in a commercial mRNA vaccine^28^.

However, not all therapeutic applications require maximal transient expression. Certain contexts require sustained, attenuated expression or tissue-specific patterns rather than ubiquitous pulse expression^30,31^. Seelig and colleagues have developed Optimus 5-Prime, which designs UTR sequences to match target expression levels or achieve cell-type-specific expression^23,25^. The approach showed robust predictive accuracy across cell types, illustrating the potential for rational design of synthetic UTRs tailored for heterologous protein expression in therapeutic contexts.

Integrated design software with graphical user interfaces (GUIs) offers distinct advantages over command-line tools and programmatic libraries for mRNA design. These platforms provide accessible, integrated environments that enable researchers across disciplines to perform the design-to-synthesis workflow. GUI-based tools facilitate rapid adoption, simplify parameter adjustment and enable real-time visualization and predictive analysis of sequences. For example, mRNAid^32^ features an intuitive interface that allows users to configure multi-objective optimization through direct parameter selection. The platform uses the DNA Chisel optimizer^33^ to implement constraint satisfaction algorithms and displays secondary structures in real time with exportable outputs. mRNAArchitect^34^, which also utilizes DNA Chisel, provides similar constraint-based optimization while adding functionality to assemble complete mRNA constructs incorporating both CDSs and UTRs. mRNAdesigner^35^ operates as a web server that processes input CDSs and produces optimized designs, automatically selecting UTR sequences from its curated database to maximize predicted translation initiation and stability when combined with the designed CDS.

We present VaxLab, an integrated platform for mRNA design that combines codon optimization, mRNA assembly, RNA feature evaluation, secondary structure analysis and sequence editing within a single interface. Unlike existing GUI platforms, VaxLab incorporates multiple state-of-the-art optimization algorithms for CDS and UTRs, enabling users to select and customize parameters according to their specific requirements. We demonstrate the platform’s capacity for rapid mRNA design and enhanced protein expression through experimental validation using influenza A virus hemagglutinin (HA) as a proof-of-concept model.

## Materials and methods

### VaxLab optimization pipeline

VaxLab includes automatic downloading and installation scripts for select publicly available optimization tools and installs selected tools on demand. When using GEMORNA-UTR, the program runs with its default settings and the user-specified UTR length (short, medium or long). For Optimus 5-Prime^23,25^ optimization, 64 synthetic 5′ UTR sequences (50 nt each) are generated, each with predicted mean ribosome load (MRL) values. The sequence with minimum offset from the user-defined target ribosome load is selected as the final 5′ UTR sequence for mRNA assembly. Simple-codon-optimizer^36^, LinearDesign^15^ and CodonBERT^20^ are executed with default options and user-defined parameters where applicable (*λ* for LinearDesign). For CUSTOM^37^ optimization, 100 synonymous CDSs are generated using the user-defined target tissue option.

For RNA secondary structure predictions, RNAfold from the ViennaRNA suite^38^ is used with default options, and secondary structure visualizations are rendered interactively using forna^39^.

### Proof-of-concept study using influenza HA

For a proof-of-concept study of the VaxLab platform, we used the HA protein from influenza A virus (strain A/California/07/2009 (H1N1), GenBank: CY121680) as the target sequence. Human β-globin (*HBB*) 5′ and 3′ UTRs (RefSeq: NM_000518.5) flanked each CDS, with a triple stop codon (UGA–UAA–UAG) inserted at the 5′ end of the 3′ UTR. Tests were performed to generate eight optimized variants using VaxLab alongside the wild-type (WT) sequence as a reference. These variants include: one using simple-codon-optimizer variant, one CUSTOM variant, five LinearDesign variants with *λ* values of 0, 2, 4, 6 and 8, and one CodonBERT variant.

### Benchmarking performance of VaxLab

A computational performance assessment was conducted in June 2025 using Google Colab Pro’s central processing unit run time. Performance metrics were continuously monitored throughout all processing steps using Python’s time.time() function and psutil.virtual_memory().used. Memory footprint was sampled at 10-s intervals, and execution time was recorded for each processing step from software installation through report generation.

Performance testing was carried out using the automated user interface testing tool Sikuli X (http://sikulix.com). To simulate first-time user conditions, the run time environment was reset before each test iteration. Following each completed run, the run time instance was disconnected and deleted, then relevant variables were updated, and the subsequent test was started with a new connection. This benchmarking protocol was performed three times.

### mRNA preparation by in vitro transcription

The DNA templates (Supplementary Table 1) was synthesized using Clonal Gene service (Twist Bioscience) supplied cloned into the pUC19 vector between the HindIII and XbaI restriction sites. PCR amplification was performed using Q5 High-Fidelity DNA Polymerase (New England Biolabs, M0492) with a forward primer (Bionics Korea) contained a T7 promoter sequence, and a reverse primer (Integrated DNA Technologies) contained a poly(dT) of 120 nucleotides and two 2′-O-methylated deoxyuridine at the 5′ end (Supplementary Table 2).

DNA fragments were eluted from 1% agarose gels using the LaboPass Gel Extraction Kit (Cosmogenetech, CMG0111) and further purified using the DNA Clean & Concentrator Kit (Zymo Research, D4014) according to the manufacturer’s instructions. DNA purity and concentration were subsequently measured using a Nanodrop spectrophotometer (Thermo).

In vitro transcription was performed using 250 ng of purified DNA template with the mMESSAGE mMACHINE T7 Transcription Kit (Invitrogen, AM1344) with 7.5 mM NTP and 6 mM CleanCap Reagent AG (3′ OMe) (TriLink Biotechnologies, N-7413) at 37 °C for 2 h. The DNA template was removed using TURBO DNase (Invitrogen, AM2239) at 37 °C for 15 min, and the RNA was purified using the RNeasy MinElute Cleanup Kit (Qiagen, 74204) and eluted with deionized water (Milli-Q). The purity and concentration of the RNA were then measured using a Nanodrop spectrophotometer (Thermo).

### Cell culture, mRNA transfection and protein extraction

HCT116 (ATCC, CCL-247) cells were cultured in McCoy’s 5A medium (WELGENE, LM005-01) supplemented with 10% fetal bovine serum (WELGENE, S001-01). Eight samples of in-vitro-transcribed RNA (500 ng each) were transfected into 1.5 × 10^5^ cells seeded in 12-well plates using Lipofectamine MessengerMAX Transfection Reagent (Invitrogen, LMRNA015) according to the manufacturer’s instructions. Medium was replaced 2 h after transfection, and cells were collected after 24 h.

Cells were washed once with ice-cold phosphate-buffered saline (PBS) and then lysed on ice. The lysis was performed using RIPA buffer (50 mM Tris pH 8.0, 100 mM NaCl, 1% NP-40 Alternative, 0.1% sodium dodecyl sulfate and 0.5% sodium deoxycholate) freshly supplemented with a suite of inhibitors. The final lysis buffer contained Protease Inhibitor Cocktail Set III (Calbiochem, 535140) diluted to 1/100, Phosphatase Inhibitor Cocktail II (AG Scientific, P-1518) diluted to 1/100, and 1 U/µl of SUPERase·In RNase Inhibitor (Invitrogen, AM2696). The stock solutions for the phosphatase inhibitor cocktails were prepared by dissolving the product in 1 ml of deionized water before dilution. Lysates were centrifuged for 30 min, and protein concentrations were determined using a bicinchoninic acid assay (Thermo, 23227).

### Western blotting

Protein samples (10 µg) were resolved on Novex Tris-Glycine Mini Protein Gel (10%, 1.0 mm, WedgeWell format; Invitrogen, XP00102BOX) at 80 V for 100 min. Proteins were transferred to 0.2-µm nitrocellulose membranes using the Trans-Blot Turbo Transfer System (Bio-Rad). Membranes were blocked for 1 h at room temperature (RT) with blocking buffer (1× PBS, 0.05% w/v Tween 20, 5% w/v skim milk) on a rocker.

Primary antibodies were applied for 2 h at RT on a rocker: GAPDH monoclonal antibody (Santa Cruz Biotechnology, sc-32233) at 1:1000 dilution in 5% skim milk, and influenza A H1N1 HA monoclonal antibody (Invitrogen, MA5-29920) at 1:2000 dilution in 1% skim milk. Membranes were washed three times with PBST (1× PBS, 0.05% w/v Tween 20) for 5 min.

Secondary antibody incubation was performed for 1 h at RT on a rocker with Peroxidase AffiniPure Goat Anti-Mouse IgG (Jackson ImmunoResearch Laboratories, 115-035-146) diluted at 1:10,000 with PBST. Following three 10-min PBST washes, protein signals were detected using SuperSignal West Pico PLUS Chemiluminescent Substrate (Thermo, 34580) according to the manufacturer’s instructions and visualized with the ChemiDoc Imaging System (Bio-Rad).

Western blot images were quantified using Image Lab software (Bio-Rad). Lanes and bands were manually designated, and then the software was used to quantify the background-adjusted intensities (adjusted volume) for bands corresponding to H1N1 HA and GAPDH.

## Results

### VaxLab provides an integrated platform for mRNA vaccine sequence design

**Fig. 1:**
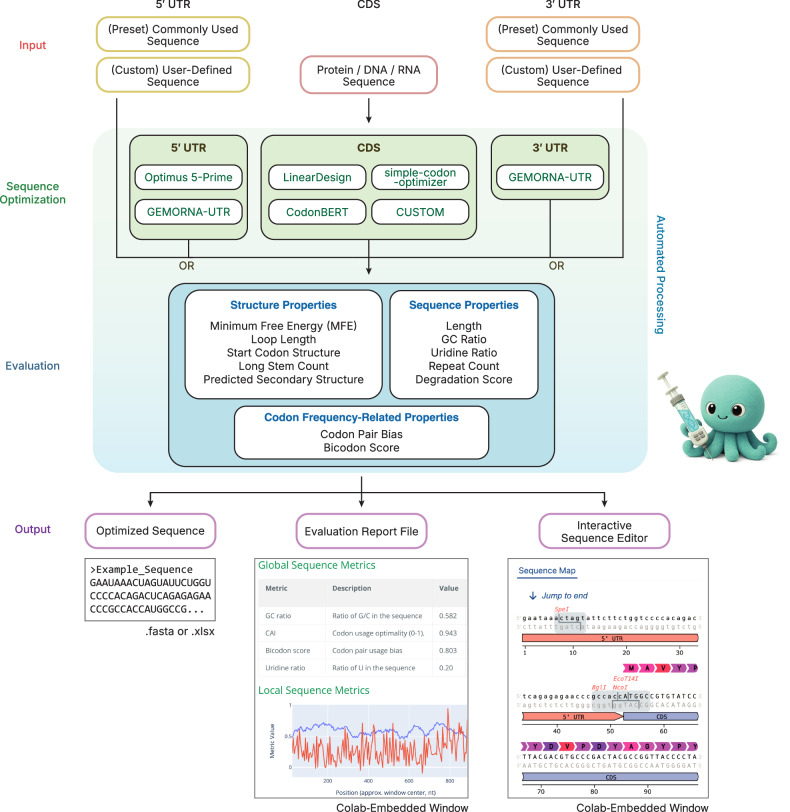
VaxLab mRNA sequence optimization workflow. Schematic representation of the VaxLab computational pipeline for optimizing mRNA sequences.

**Fig. 2:**
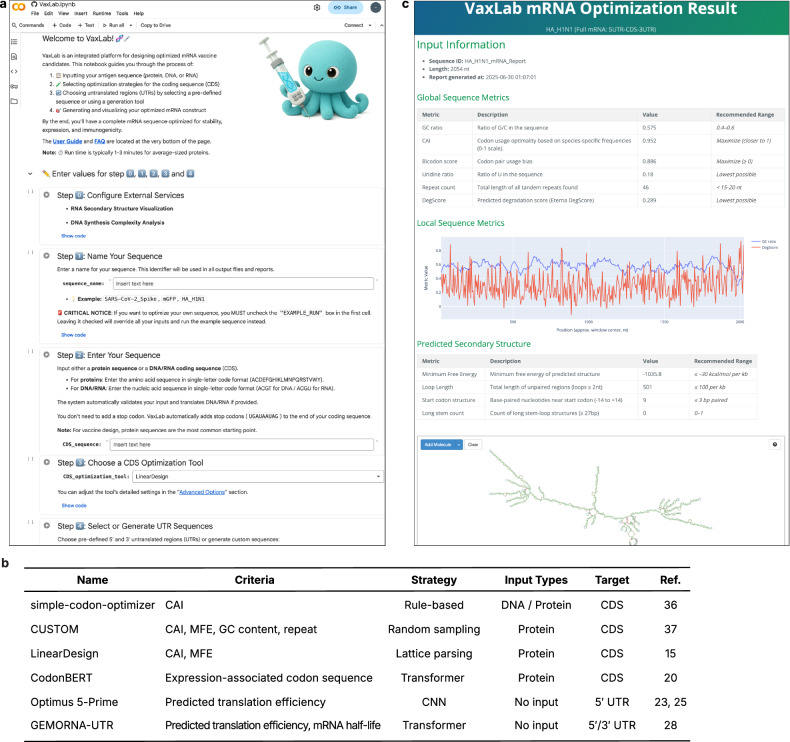
VaxLab user interface and list of integrated tools. **a** VaxLab notebook interface showing the initial settings screen. **b** Overview of five optimization tools integrated in VaxLab, showing objective functions, algorithmic approaches, input requirements and target regions. **c** Example optimization report for influenza A virus (IAV) HA mRNA processed with LinearDesign. The report displays summary metrics, local GC content, uridine distribution, predicted structure features and secondary structure visualization.

VaxLab automates the entire mRNA vaccine sequence design workflow through a web-based interactive notebook environment (Fig. 1). The platform addresses practical barriers in mRNA optimization by consolidating multiple design tools within a single, accessible environment (Fig. 2a). The system accepts amino acid, DNA or RNA sequences as input and routes them to user-selected optimization tools. For the UTR regions, users can either upload their own custom sequences or select from built-in libraries of human-derived sequences that include five options for the 5′ UTR and seven options for the 3′ UTR. These predefined libraries feature sequences from clinically proven vaccines, including those developed by Pfizer/BioNTech and Moderna. For more specialized applications, users can generate synthetic UTRs using tools such as Optimus 5-Prime, which enables tissue-specific expression or defined translation levels^25^, or GEMORNA-UTR^28^ for other custom requirements.

VaxLab offers multiple approaches for codon optimization of the CDS (Fig. 2b). It includes tools based on codon usage profiles (simple-codon-optimizer^36^ and CUSTOM^37^), combined secondary structure and codon usage optimization (LinearDesign^15^), and language model-based expression prediction (CodonBERT^20^). While codon usage-based tools typically yield moderate protein production across contexts, the structure-aware methods may provide superior results for therapeutic applications by enhancing RNA stability and reducing innate immune response, as demonstrated in mouse studies with severe acute respiratory syndrome coronavirus 2 (SARS-CoV-2) and varicella-zoster virus vaccines^15^. This tool diversity enables selection of optimal strategies for specific objectives.

While each integrated algorithm has been validated in prior studies, their combined effects with other mRNA vaccine components, such as UTR sequences or lipid nanoparticle (LNP) formulations, remain largely unexplored. VaxLab is designed to address this gap by allowing users to generate and evaluate multiple candidate combinations, facilitating the discovery of optimal designs. To guide decision-making among design alternatives, VaxLab calculates ten predictive metrics encompassing critical aspects of mRNA performance. These metrics include codon adaptation index (CAI)^40^, codon pair bias^41^, minimum free energy (MFE), RNA degradation score (DegScore)^12^, RNA secondary structure properties and uridine frequency as parameters indicative of RNA stability and protein production^12,14^. Additional features such as GC content, tandem repeat counts and repeat ratio inform manufacturing feasibility.

The comprehensive evaluation report includes interactive RNA secondary structure visualization via forna^39^ (Fig. 2c). The integrated vector editor by Teselagen^42^ enables elimination of restriction sites that interfere with manufacturing or modification of sequences for cloning vector compatibility. VaxLab exports final designs in standard FASTA or GenBank formats, as well as prefilled spreadsheets compatible with commercial synthesis services, eliminating manual data entry errors.

### CDS optimization strategies differentially impact sequence properties

We tested VaxLab’s workflow by designing nine different CDSs for influenza HA protein using four optimization strategies. All optimized sequences incorporated human β-globin (*HBB*) 5′ and 3′ UTRs, which are commonly used in mRNA vaccine studies.

**Fig. 3:**
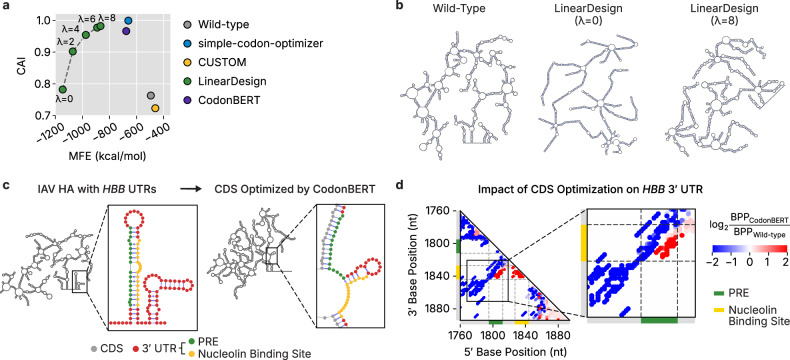
Comparative analysis of optimized HA mRNAs from different tools. **a** CAI and MFE of WT and optimized full-length mRNA sequences for the IAV HA mRNAs with human β-globin (*HBB*) UTRs. LinearDesign variants are shown with different *λ* parameter values. **b** Predicted secondary structures of WT and LinearDesign-optimized HA mRNAs with *HBB* UTRs, computed using RNAfold. **c** Full-length secondary structure predictions for WT and CodonBERT-optimized HA mRNAs with *HBB* UTRs. PRE and nucleolin binding site regions are highlighted with colors. **d** Base-pairing probability changes between WT and CodonBERT-optimized HA mRNAs with *HBB* UTRs. Heatmap shows log₂ ratios, with blue indicating decreased pairing and red indicating increased pairing in optimized constructs. PRE and nucleolin binding site positions are marked with green and yellow bars within *HBB* 3′ UTR according to Jiang et al.^43^.

All optimization methods except one achieved improved sequence characteristics in terms of codon usage and secondary structure properties (Fig. 3a). Simple-codon-optimizer produced the highest CAI values (1.0) by replacing all codons to the most frequently used variants. CUSTOM generated lower CAI values (0.72), probably because it targets tissue-specific codon usage patterns that differ from the genome-wide frequencies used in standard CAI calculations. LinearDesign’s lambda (*λ*) parameter controlled the balance between codon usage and secondary structure optimization. Increasing *λ* from 0 to 8 improved CAI from 0.78 to 0.98, while MFE values increased from −1,146 to −866 kcal mol^−1^. CodonBERT prioritized CAI (0.97) over RNA folding (MFE −676 kcal mol^−1^), producing sequences with characteristics similar to those from simple-codon-optimizer.

Secondary structure predictions revealed distinct folding patterns for each optimization tool (Fig. 3b and Supplementary Fig. 1). LinearDesign sequences optimized with *λ* = 0 exhibited extensive base pairing for structural stability, while those with *λ* = 8 displayed relaxed structures with numerous internal loops and multiloops (Fig. 3b).

We investigated whether CDS optimization affects the folding of conserved regulatory elements within the *HBB*-derived 3′ UTR. This UTR is known to contain two functional *cis*-regulatory motifs, a pyrimidine-rich element (PRE) and a nucleolin binding site, embedded within a conserved stem–loop structure^43^ (Fig. 3c, left). This structure recruits RNA-binding proteins PCBP1 (αCP1) and nucleolin, which protect transcripts from degradation^43^. When paired with the WT HA CDS, the regulatory stem–loop structure remained unchanged, preserving both PRE and nucleolin binding regions (Fig. 3c, d). By contrast, codon optimization substantially altered the structural ensemble around the conserved motif structure. CodonBERT caused the most severe disruption by destabilizing the original stem–loop structure and creating extensive CDS–3′ UTR interactions (Fig. 3d), while other optimization tools strengthened the lower stem structure but weakened the upper part (Supplementary Fig. 2). These results show that CDS optimization can unintentionally damage regulatory structures within the 3′ UTR, potentially destabilizing the mRNA. This highlights the importance of computational validation of each design to identify potentially compromised candidates before use.

### VaxLab evaluates synthesis and manufacturing feasibility

Optimized sequences require synthesis through oligonucleotide synthesis and long DNA assembly to generate templates for RNA production^44^. Synthesis failure of long DNA fragments can be predicted from intrinsic sequence properties, including extreme GC content, repeated sequence patterns and stable secondary structures^45^. Such sequences force more complex and error-prone assembly processes, increasing production time and risk^45^. To mitigate these challenges, VaxLab reports the synthesis complexity of designed sequences calculated using an application programming interface from Integrated DNA Technologies. This assessment enables users to prevent complications by adjusting sequences to improve synthesis feasibility.

**Fig. 4:**
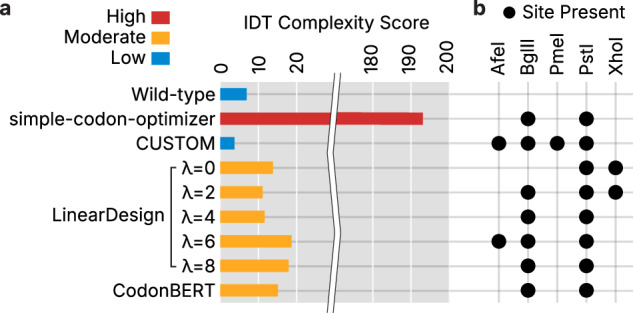
Evaluation of manufacturing compatibility for optimized HA mRNAs. **a** Integrated DNA Technologies (IDT) complexity scores for WT and optimized sequences across optimization strategies, including LinearDesign variants with different *λ* values. Complexity classifications (low, moderate and high) are based on IDT’s internal criteria. **b** Matrix summarizing the presence (black) or absence (white) of restriction enzyme recognition sites across WT and optimized HA CDSs.

Candidate sequences for the HA protein demonstrated a wide range of synthesis difficulties (Fig. 4a). The sequence optimized by simple-codon-optimizer was predicted to be the most difficult to synthesize due to highly repetitive nucleotide patterns. Even short motifs of three repeated amino acids generate nine-nucleotide repeats when CAI is maximized. Synthesis complexity was lowest when constraints were relaxed or balanced between high CAI and low MFE (Fig. 4a).

VaxLab also detects unintended restriction enzyme sites within CDSs that can disrupt modular assembly or template linearization during manufacturing^46^. The platform screens optimized sequences against 533 restriction enzymes and reports results through an integrated sequence editor for site removal or modification. In our test HA protein designs, five enzymes commonly used in mRNA vaccine construction (AfeI, BglII, PmeI, PstI and XhoI) were absent from WT sequence but introduced by many optimization algorithms (Fig. 4b). Notably, all optimization methods consistently introduced the PstI recognition site (CTGCAG). This site corresponds to the most frequently used codons for leucine (CTG, 41% usage) and glutamine (CAG, 75% usage) in human mRNAs. These cases demonstrate that codon optimization strategies impose distinct codon usage patterns that inadvertently generate restriction sites (Supplementary Fig. 3a,b), highlighting the importance of post-optimization sequence validation and editing.

In mRNA platforms, payload length and content vary considerably depending on the application. VaxLab’s feasibility reports enable users to consider synthesis feasibility alongside other performance factors when selecting tools or parameters for variable payload conditions. The integrated sequence editor provides a convenient environment for manually eliminating problematic sites through synonymous codon substitutions (Fig. 1, bottom right). The integrated editor includes standard cloning tools such as primer design and sequence manipulations, enabling users to prepare synthesis-ready constructs.

### Sequence optimization exhibits rapid, memory‑efficient performance with VaxLab

We evaluated VaxLab computational performance across two dimensions: total run time and peak memory usage under Google Colab notebook constraints. VaxLab’s run time and memory usage depend on the selected tool workflow and protein sequence length. For instance, LinearDesign’s computational and memory complexity increases quadratically with sequence length^15^. Efficient execution accelerates the design-evaluation cycle, enabling faster identification of optimal candidate sequences. This efficiency is particularly valuable for time-sensitive applications such as outbreak pathogen vaccines and personalized cancer vaccines^3,47^.

**Fig. 5:**
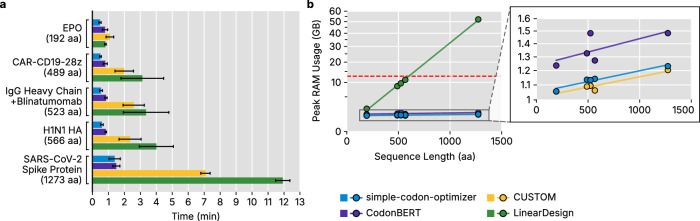
VaxLab benchmark results for total running time and memory footprint. **a** Total execution time by protein sequence and optimization tool. Values represent the mean and standard deviation from three independent runs. **b** Peak RAM usage versus input sequence length for each optimization tool. Linear regression lines are fit to data points. The dashed red line indicates Google Colab free tier memory limit (12.7 GB). Inset magnifies overlapping data points for clarity.

We measured total run times for all 20 combinations of 4 CDS optimization tools and 5 representative proteins (Fig. 5a and Supplementary Tables 3 and 4). The test proteins included human erythropoietin (EPO; 192 amino acids), influenza A H1N1 HA (566 amino acids), SARS-CoV-2 spike glycoprotein (1273 amino acids), a blinatumomab variant with cleavable IgG heavy chain (523 amino acids) and a CD19-specific 1928z chimeric antigen receptor (CAR) (489 amino acids). All optimizations completed within 13 min, with CDS optimization comprising 43% of total run time on average. Setup overhead was negligible (~22 s).

Total run time scaled with protein length. EPO optimization averaged 47 s (fastest: 28 s with simple-codon-optimizer), while SARS-CoV-2 spike optimization averaged 5 min 28 s (slowest: 11 min 55 s with LinearDesign). VaxLab generated an average of 55 sequence variations per hour for different tool combinations or optimization parameters. After initial installation and setup, VaxLab throughput depends completely on the computational complexity of the selected CDS algorithm and sequence length.

Peak memory consumption was measured for the same 20 tool–protein pairs (Fig. 5b). Memory consumption scales linearly (simple-codon-optimizer, CUSTOM, CodonBERT) or quadratically (LinearDesign) with protein length (Fig. 5b). Google Colab’s free tier provides 12.7 GB random-access memory (RAM) as of June 2025. Only one workload combination exceeded this limit: LinearDesign applied to SARS-CoV-2 spike, which peaked at 52 GB. LinearDesign processes sequences up to 566 amino acids within the 12.7 GB limit, but longer proteins require alternative optimizers or sequence segmentation. All other tools completed optimization of proteins up to 1273 amino acids within Colab’s memory limit, allowing VaxLab implementation in commodity cloud environments for most applications.

### Optimization strategy selection determines protein expression enhancement in VaxLab-optimized mRNA

**Fig. 6:**
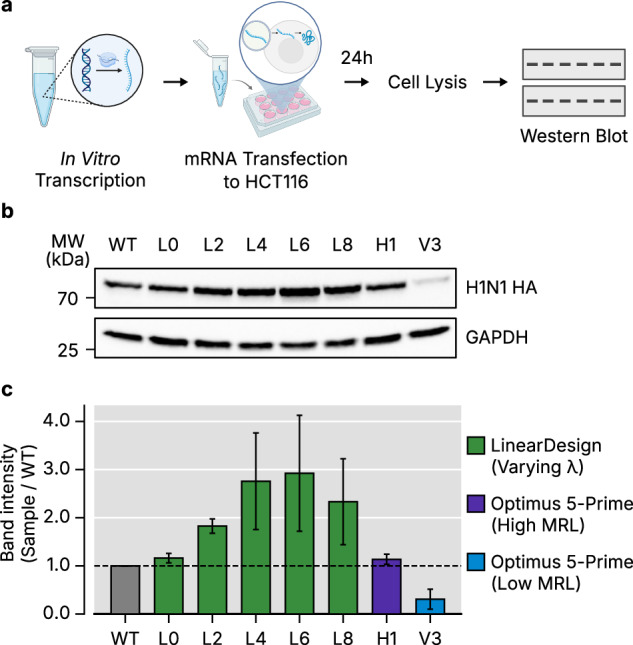
Protein expression of VaxLab-optimized sequences in cells. **a** Experimental workflow for validating intracellular protein production of optimized HA mRNAs. **b** Western blot analysis of HA protein expression in HCT116 cells 24 h after transfection with the VaxLab-optimized mRNAs. **c** The bar plot shows the quantification of H1N1 HA band intensity, normalized to GAPDH, in the sample relative to the WT. Data are presented as the mean ± standard deviation of three independent replicates.

We quantified intracellular HA protein production after transfecting cells with seven VaxLab-optimized mRNAs (five CDS variants and two 5′ UTR variants) and a WT control (Fig. 6a). The control contained native *HBB* 5′ and 3′ UTRs, a WT viral HA CDS, and a 120-nt poly(A) tail. Among the seven VaxLab-optimized mRNAs, five contain the CDSs generated from LinearDesign with different CAI-MFE weight parameters *λ* = 0, 2, 4, 6 or 8 (L0–8) with *HBB* 5′ and 3′ UTRs. The CDSs of other two (H1 and V3) were the same as the CDS of L4, but the 5′ UTR sequences were replaced with the one generated using Optimus 5-Prime for either high (H1; predicted MRL of 9.76) or low (V3; predicted MRL of 3.50) translational efficiency. We collected cells 24 h after transfection and measured HA levels by western blotting.

All VaxLab-optimized CDSs increased HA expression relative to WT except V3, whose low-MRL 5′ UTR suppressed translation (Fig. 6b, c and Supplementary Fig. 4). Synthetic 5′ UTRs substantially affected protein production: H1 produced 3.7-fold more protein than V3, closely matching the 2.8-fold difference reported previously^25^. However, H1 underperformed L4, which shares the same CDS but differs in 5′ UTR. This shows that combining individually favorable elements does not guarantee maximal expression and suggests the competence of the *HBB* 5′ UTR. Among CDS variants, L6 with balanced CAI-MFE yielded the highest output (2.9-fold gain over WT) compared with CAI-focused variant L8 or MFE-focused variant L0.

These observations reflect the multifactorial nature of mRNA translation. Interactions among the 5′ UTR, CDS, 3′ UTR and poly(A) tail—as well as their influence on RNA-binding protein recruitment—complicate purely rational design approaches^8–10^. Manufacturing variability introduces additional uncertainty. In vitro transcription efficiency varies with sequence composition and can introduce double-stranded RNA impurities that induce unfavorable cellular responses for protein production^48–50^. Consequently, moderate-scale empirical screening coupled with initial computational optimization remains essential.

## Discussion

mRNA sequence design requires balancing translation efficiency, RNA stability and manufacturability. The optimal balance varies with therapeutic context, target tissue and antigen properties^8–10^. Because no single algorithm consistently outperforms others, researchers routinely test several optimization strategies. However, existing tools operate in isolation, rely on disparate installation procedures and require users to understand algorithmic characteristics to tune parameters effectively. These barriers hinder active exploration and evaluation of emerging tools that leverage advances in RNA biology and artificial intelligence.

VaxLab centralizes these workflows by providing a browser-based interface to codon optimizers that otherwise demand extensive local configuration. Users can optimize CDSs and UTRs separately or jointly, then compare designs using a standardized set of ten predictive metrics encompassing translation efficiency (CAI and codon-pair bias), RNA stability (MFE and secondary structure scores), and manufacturing constraints (GC content and synthesis complexity). Standardized reports enable side-by-side evaluation without manually matching result formats from different programs.

The platform provides interactive visualization of predicted secondary structure with highlighted regions critical for mRNA performance. This feature enables rapid inspection of Kozak motifs, ribosome-binding sites and stem–loop structures within stabilizing *cis*-acting elements^43,51^. Changes in these structures can affect translation initiation, elongation and RNA stability^12,52^. Exposed single-stranded regions become more susceptible to endonuclease cleavage^53,54^, while long double-stranded RNA structures can trigger innate immune responses through pattern recognition receptors such as RIG-I and MDA5^55,56^. Integrated visualization helps users detect potential interference before experimental testing.

To streamline manufacturing, VaxLab reports synthesis complexity for designed sequences and flags problematic motifs, including restriction sites and long homopolymers. An integrated sequence editor allows immediate correction. On standard cloud infrastructure, the complete pipeline generates synthesis-ready sequences for proteins up to 500 amino acids within 5–13 min, whereas equivalent manual workflows require several hours to days. Industrial biofoundries increasingly depend on machine-readable sequence standards and automated design-to-synthesis pipelines. VaxLab accepts sequences in standard formats and exports to FASTA, GenBank and commercial provider formats such as Twist Bioscience’s.

Although benchmarking focused on a viral vaccine antigen, the same architecture supports protein-replacement therapies, antibody expression and genome-editing cargos, each imposing distinct design constraints. Replacement therapies often require sustained expression with minimal immune activation^57^, while genome editors demand robust yet controlled expression to avoid off-target effects^58^. By reporting multiparameter scores rather than single rankings, VaxLab allows users to navigate trade-offs systematically.

The modular codebase of VaxLab permits rapid incorporation of emerging algorithms in the future. Potential additions include generative-model codon optimizers, more UTR design tools for distinct capabilities, signal-peptide insertion^59^ from predefined libraries, automated implementation of stabilizing substitutions (for example, 2P or HexaPro for SARS-CoV-2 vaccines^60,61^), multiprotein designs with optimized linkers^62^, and filters that eliminate frameshifting or unintended cleavage sites^63,64^. VaxLab provides a comprehensive design platform for the accelerated development of mRNA applications.

## Supplementary information

Supplementary Information

Supplementary Tables 1–4

## Data Availability

VaxLab can be accessed at https://colab.research.google.com/github/ChangLabSNU/VaxLab/blob/main/VaxLab.ipynb.
