# Supplementary material for: VaxLab: integrated platform for rapid multistrategy mRNA vaccine design
Source: Exp Mol Med. 2026 Apr 8;58(4):1149–57. doi: 10.1038/s12276-026-01637-y (PMC13144369; doi:10.1038/s12276-026-01637-y)
Supplement: Supplementary file 1 — Supplementary Information [file 12276_2026_1637_MOESM1_ESM.pdf]

Supplementary Information

VaxLab: Integrated Platform for Rapid Multi-Strategy mRNA Vaccine Design

Junsoo Kim[1,2,4], Yoojung Han[1,2,4], Chae Young Kwon[1], and Hyeshik Chang[1,2,3,*]

[1] Center for RNA Research, Institute for Basic Science, Seoul National University, 08826, Republic of Korea

[2] Interdisciplinary Program in Bioinformatics, Seoul National University, Seoul, 08826, Republic of Korea

[3] School of Biological Sciences, Seoul National University, Seoul, 08826, Republic of Korea

[4] These authors contributed equally to this work.

* Correspondence: hyeshik@snu.ac.kr (H.C.)

Supplementary Figure 1. Predicted secondary structures of influenza A HA mRNAs optimized using different CDS optimization strategies

Supplementary Figure 2. Differential effects of CDS optimization strategies on HBB 3′ UTR regulatory structure

Supplementary Figure 3. Codon usage patterns and restriction site analysis across CDS optimization strategies

Supplementary Figure 4. Additional western blots from the cells transfected with the HA-coding optimized mRNA sequences

(The supplementary tables have been provided as a separate Excel file.)

Supplementary Table 1. List of PCR primer sequences used to prepare the DNA template for in vitro transcription

Supplementary Table 2. List of protein sequences used for benchmarking

Supplementary Table 3. Execution time and memory usage depending on the protein and CDS optimization tool

Supplementary Table 4. An optimized sequence list for coding Influenza A HA used in the validation experiment

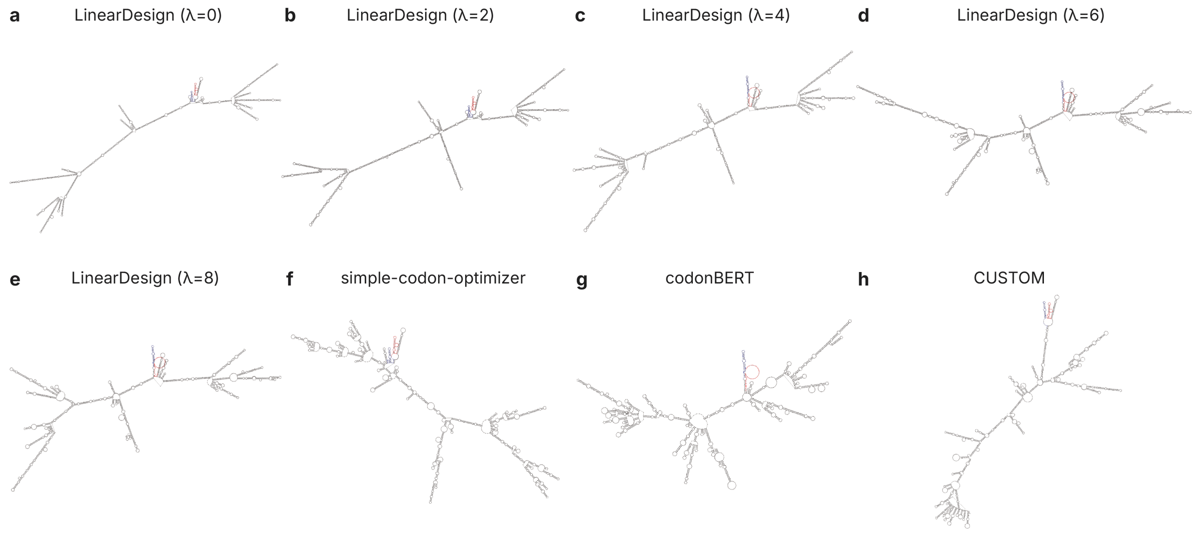

Supplementary Figure 1. Predicted secondary structures of influenza A HA mRNAs optimized using different CDS optimization strategies

Predicted secondary structures computed using RNAfold. (a–e) LinearDeisgn optimization with varying λ parameters (λ = 0, 2, 4, 6, 8). (f) Simple-codon-optimizer. (g) CodonBERT. (h) CUSTOM. All sequences include identical HBB-derived 5′ and 3′ UTRs.

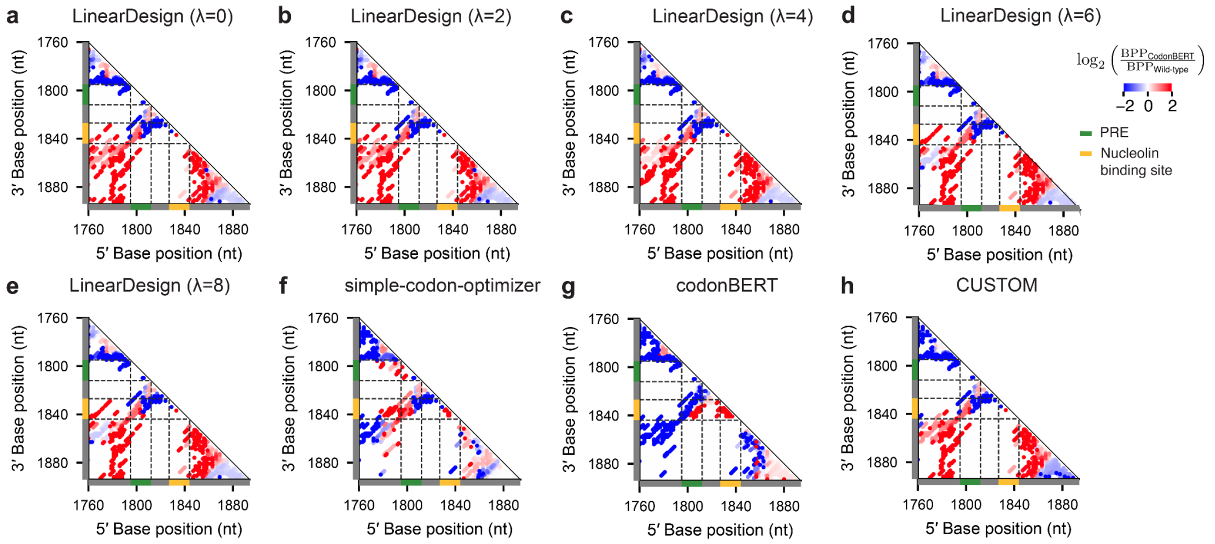

Supplementary Figure 2. Differential effects of CDS optimization strategies on *HBB* 3′ UTR regulatory structure

Heat maps showing base-pairing probability changes as $\log_2$ ratios of optimized versus wild-type sequences for different CDS optimization methods: (a-e) LinearDesign with λ=0, 2, 4, 6, 8; (f) simple-codon-optimizer; (g) CodonBERT; (h) CUSTOM. Blue indicates decreased pairing and red indicates increased pairing in optimized constructs. Pyrimidine-rich element (PRE) and nucleolin binding site positions are marked with green and yellow bars within *HBB* 3′ UTR according to Jiang *et al.*[1]

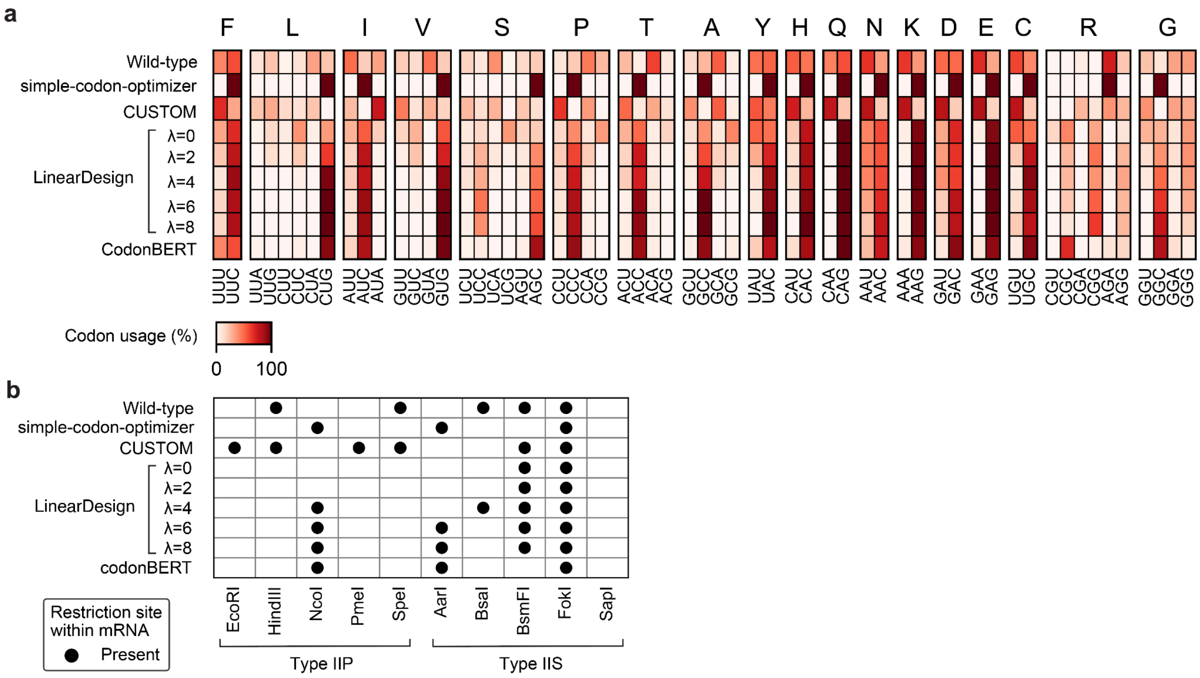

Supplementary Figure 3. Codon usage patterns and restriction site analysis across CDS optimization strategies

(a) Heat map showing codon usage for each amino acid across wild-type and optimized HA sequences. Methionine and tryptophan, which are encoded by single codons, as well as stop codons, were excluded from this analysis. (b) Matrix indicating the presence (black dots) or absence (white) of restriction enzyme recognition sites in optimized HA coding sequences.

Replicate 2

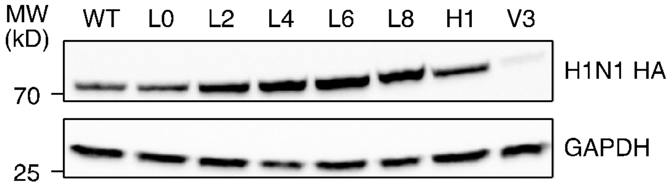

Replicate 3

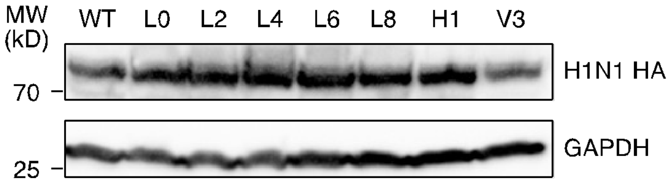

Supplementary Figure 4. Additional western blots from the cells transfected with the HA-coding optimized mRNA sequences

Western blot replicates data of Figure 6b.

(The supplementary tables have been provided as a separate Excel file.)

Supplementary Table 1. List of PCR primer sequences used to prepare the DNA template for in vitro transcription

List of two forward primers and one reverse primer used for the PCR experiments. Both forward primers include a T7 promoter sequence, and 'mU' denotes 2′-O-methylated deoxyuridine.

Supplementary Table 2. List of protein sequences used for benchmarking

List of five protein sequences used to benchmark the execution time and RAM usage of VaxLab.

Supplementary Table 3. Execution time and memory usage depending on the protein and CDS optimization tool

Triplicate measurements of the execution time for each process of VaxLab (a–c) and the RAM usage measured at 10-second intervals during execution (d–f).

Supplementary Table 4. An optimized sequence list for coding Influenza A HA used in the validation experiment

The DNA template sequences used to generate a total of eight mRNAs, including the wild-type (WT), for a validation experiment to confirm protein expression levels. For each entry, the sequence for CleanCap, the specific sequences for the 5′ UTR, CDS, and 3′ UTR, and the full-length sequence are provided. The designations "HBB-5'UTR" and "HBB-3'UTR" refer to the native UTR sequences from the wild-type human β-globin *(HBB)* gene. "LD" indicates that the sequence was optimized using LinearDesign, with the subsequent number representing the lambda value used as an option. "Optimus5prime" refers to a sequence previously generated with that model and used in a prior study[2].

Reference

1. Jiang, Y., Xu, X. S. & Russell, J. E. A nucleolin-binding 3' untranslated region element stabilizes beta-globin mRNA in vivo. *Mol. Cell. Biol.* **26**, 2419–2429 (2006).

2. Castillo-Hair, S. *et al.* Optimizing 5’UTRs for mRNA-delivered gene editing using deep learning. *Nat. Commun.* **15**, 5284 (2024).
